# Case report: Infective endocarditis after transcatheter aortic valve implantation surgically treated with sutureless prosthesis and ascending aorta replacement

**DOI:** 10.3389/fcvm.2023.1194304

**Published:** 2023-08-21

**Authors:** Francesco Cabrucci, Beatrice Bacchi, Riccardo Codecasa, Pierluigi Stefàno

**Affiliations:** Department of Cardiac Surgery, Careggi University Hospital, Florence, Italy

**Keywords:** endocarditis, TAVI, porcelain aorta, sutureless prosthesis, hostile aortic root

## Abstract

Infective endocarditis on transcatheter aortic valve implantation (TAVI) represents an increasingly frequent challenge for cardiac surgeons. Patients undergoing TAVI usually have high mortality risk scores and unsuitable anatomy for the traditional surgical approach. Therefore, surgical planning is crucial, albeit sometimes intraoperative findings can be unexpected and arduous. We describe a case of infective endocarditis on TAVI in a patient with a porcelain aorta and “hostile” aortic root surgically treated with Perceval sutureless prosthesis and ascending aorta replacement.

## Introduction

As transcatheter aortic valve implantation (TAVI) becomes more popular, the management of device failure will be progressively more frequent (1). Among long-term complications, infective endocarditis on TAVI (TAVI-IE) is a challenging issue for cardiac surgeons, with an incidence of 0.2%–3.1% at 1 year (2), leading to heart failure in 37.1% of cases (3). Although early surgery in patients with native valve endocarditis has demonstrated survival advantages, the therapeutic options in prosthesis valve endocarditis (medical management vs. surgery) are still debated. In addition, considering that patients with TAVI-IE are a very high-risk cohort in which no clear benefits are observed after cardiac surgery vs. medical therapy alone (4), the management should be individualized based on the severity of endocarditis and guided by a dedicated team (5).

Despite the approach chosen, the survival of patients with TAVI-IE is generally poor, and about one-fourth of them (22%) have undergone surgical explantation of the device (6). We report a case of surgical treatment of a severe TAVI-IE in a high-risk patient with a porcelain ascending aorta.

## Case description

An 81-year-old woman with hypertension, dyslipidemia, and rheumatoid arthritis underwent transfemoral-TAVI (CoreValve-Evolute Pro, 23 mm, Medtronic, Minneapolis, MN, USA) for aortic stenosis in May 2020 after being evaluated by the heart team as a high-risk patient with an society of thoracic surgeons (STS) predicted risk of mortality (PROM) of 4.39%.

**Figure 1:**
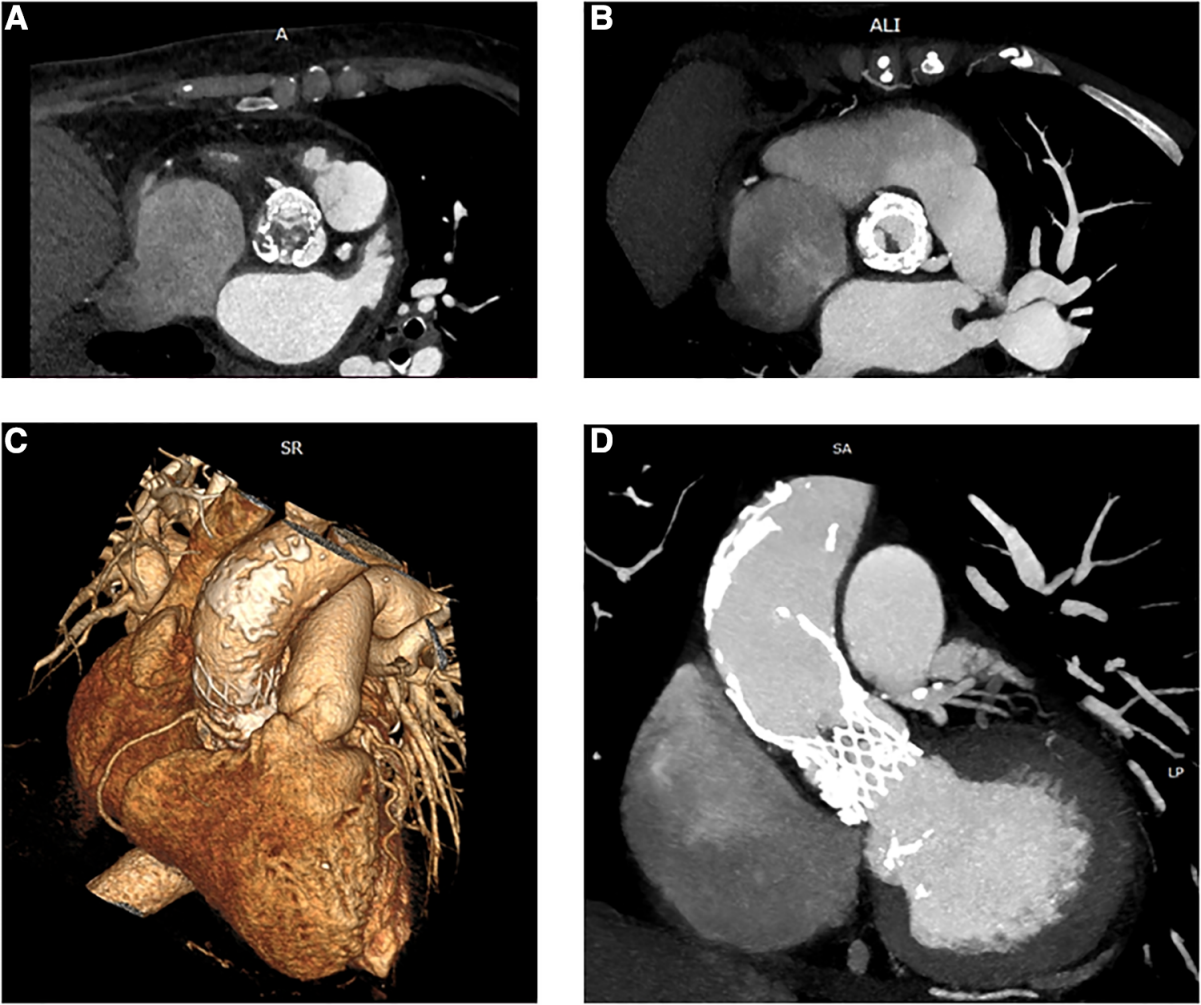
Preoperative CT: axial view of the CoreValve prosthesis with vegetations in the left (**A**) and right (**B**) coronary cusps. 3D reconstruction of the ascending aorta with features of porcelain aorta (**C**). Multiplanar reconstruction (MPR) shows thrombosis and vegetation inside the CoreValve scaffold and prominent calcification of the aorta (**D**).

**Figure 2:**
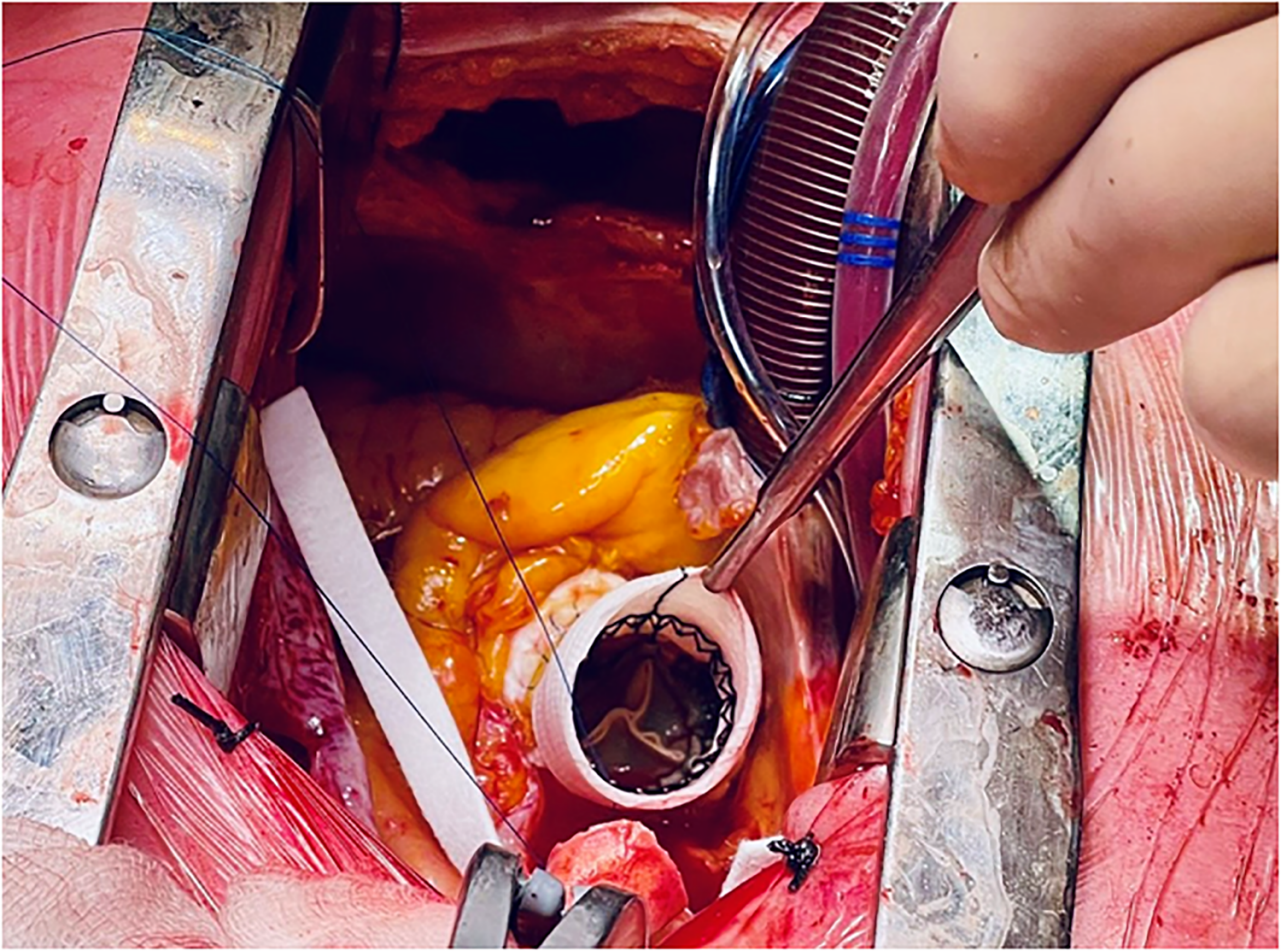
Perceval S sizer inside the 24-mm Dacron graft with one guide stitch.

In November 2021, she was admitted to our emergency department due to fatigue, exertional dyspnea, and intermittent fever for 3 weeks, treated with empirical antibiotics. Electrocardiography demonstrated sinus rhythm, 70 beats/min, with features of left ventricle hypertrophy. The blood sample revealed moderate leukocytosis and 1.14 μg/L procalcitonin. Blood cultures were positive for *Staphylococcus aureus*. Both transthoracic echocardiogram (TTE) and transesophageal echocardiography (TEE) revealed large, mobile vegetation on the aortic prosthesis and non-structural valve dysfunction (NSVD) with a mean gradient of 54 mmHg. Angio-CT scanning showed thrombosis between the left coronary and non-coronary cusps of the prosthesis (Figure 1) and splenic embolization. Antibiotic therapy was started, guided by an antibiogram. Despite initial clinical improvement, 8 days after hospitalization, the patient developed a fever recurrence and initial hemodynamic deterioration requiring intensive care unit admission. Although the patient was previously discarded for surgery, TAVI-IE at this time was considered a surgical indication, despite the very high-risk score (STS PROM: 10.38%). After a full sternotomy, cardiopulmonary bypass (CPB) was set using the right axillary artery (by the interposition of a Dacron graft) and the right atrium. Aorta was clamped (in the upper zone 0), and Del Nido cardioplegia was administered. Through transverse aortotomy, the prosthesis was excised with the native aortic valve. The prosthesis was entirely covered by fibrotic tissue, and signs of endocarditis were also detected. Moreover, between the last part of the Valsalva sinus and the sinotubular junction, the aortic lumen was narrowed by a full-thickness circumferential calcified shelf, in this case described as an aortic sinotubular ridge. Due to this challenging anatomy, only the Perceval (Livanova, London, UK) S (small) sizer was suitable. In addition, considering the extremely calcified aorta and coronary ostia, the option to perform a Bentall-De Bono or an enlargement of the aortic root procedure was judged hazardous. Therefore, first, ascending aorta was replaced with a 24-mm Dacron tube to allow safe deployment of the sutureless valve, Perceval S, followed by ballooning. In addition, the aortic sinotubular ridge thickening did not allow placing the three standard guide stitches used for Perceval deployment. Therefore, only one guide stitch was used to parachute down the prosthesis (Figure 2). No extra stitches were used to anchor the valve to the Dacron graft. CPB was easily discontinued under TEE monitoring, demonstrating that the prosthesis was well-functioning. The explanted prosthesis was sent for microbiology analysis, and the result showed growth only for Gram-positive aerobic pathogens. The patient was extubated after 6 h and discharged to rehab on postoperative day 20. After 15 months of follow-up, no negative events were reported. Follow-up TTE showed preserved left ventricular ejection fraction (LVEF) and a well-functioning aortic prosthesis (mean gradient of 19 mmHg, no regurgitation).

## Discussion

Undoubtedly, TAVI brought a valid alternative for treating many patients affected by aortic stenosis. Transcatheter prostheses may need explantation due to prosthetic endocarditis (the most common reason), SVD, early paravalvular leakage, and prosthesis–patient mismatch (7). In the case of endocarditis, in addition to TAVI's explant, various possible combined cardiac interventions confer significant perioperative mortality and morbidity.

Therefore, the exact diagnosis and localization of endocarditis are strictly required to correctly program the surgical intervention (8). Prosthetic valve shadow may obscure smaller vegetations and smaller abscesses at the echocardiographic examination (9); thus, preoperative contrast-enhanced multislice CT or 18F-FDG PET/CT should be performed.

In addition, the evaluation of the aortic root morphology and dimensions, the degree of calcification, and the coronary ostia is paramount. The aortic annulus and root were narrow and extremely calcified in this case. Moreover, since the TAVI was performed more than 1 year before, the prosthesis had undergone re-endothelialization and caused narrowing of the aortic sinotubular ridge. In addition, an active inflammatory process due to endocarditis was present on the prosthesis. All these aspects made the aortic root very difficult to deal with.

In this scenario, a sutureless valve, as previously described (10), may be particularly useful since, in case of a hostile aortic root, it avoids the placement of suture stitches, preventing possible tissue tearing tissue and sparing the need for root replacement. Moreover, in the case of endocarditis, the prosthesis structure made only by leaflets mounted onto the stent may contribute to reducing the infectious processes. In addition, considering that TAVI should be reserved for high-risk patients, the significant reduction of cross-clamp and CPB time associated with a sutureless valve is fundamental in this special cohort. No increased risk of device dislocation, paravalvular leakage, the occurrence of third-degree atrioventricular block, or the need for postoperative permanent pacemaker implantation has been described with a sutureless valve after infective endocarditis (10). However, this technique is subject to all limitations inherent to a small cohort of patients and the need for long-term follow-up.

In conclusion, in the case of TAVI-IE associated with a “hostile” (calcified and narrowed) aortic root, a combination of segmental aortic replacement and sutureless prosthesis implantation may avoid the placement of annular stitches, possible annular tearing, and the need for root replacement.

## Data Availability

The original contributions presented in the study are included in the article, further inquiries can be directed to the corresponding author.
